# Ruxolitinib as an Effective and Steroid-Sparing First-Line Treatment in Newly Diagnosed BOS Patients After Hematopoietic Stem Cell Transplantation

**DOI:** 10.3389/fphar.2022.916472

**Published:** 2022-07-05

**Authors:** Xiaoyu Zhang, Xiaoli Zhao, Yuyan Shen, Yuanyuan Shi, Lining Zhang, Mengze Hao, Fei Zhao, Rongli Zhang, Jialin Wei, Sizhou Feng, Yi He, Erlie Jiang, Mingzhe Han

**Affiliations:** State Key Laboratory of Experimental Hematology, Department of Hematopoietic Stem Cell Transplantation, National Clinical Research Center for Blood Diseases, Haihe Laboratory of Cell Ecosystem, Institute of Hematology and Blood Diseases Hospital, Chinese Academy of Medical Sciences and Peking Union Medical College, Tianjin, China

**Keywords:** bronchiolitis obliterans syndrome (BOS), ruxolitinib, hematopoietic stem cell transplantation (HSCT), cGVHD, pulmonary function test (PFT)

## Abstract

Bronchiolitis obliterans syndrome (BOS) is a life-threatening pulmonary complication of chronic graft-versus-host disease (cGVHD) after allogeneic hematopoietic stem cell transplantation (HSCT). In this study, we retrospectively identified seven patients newly diagnosed with BOS post HSCT and analyzed the outcomes in those patients treated with ruxolitinib as a first-line treatment. All seven patients achieved symptom responses within 2 weeks after ruxolitinib administration. Three months after treatment, five patients (71.43%) achieved a CR, and two (28.57%) achieved a PR. The overall response rate (ORR) was 100%. In addition, the steroid therapy was determined within 2 months after ruxolitinib treatment, indicating ruxolitinib as a steroid-sparing agent. We also found that ruxolitinib was well-tolerated and safe in treating newly diagnosed BOS. According to our results, ruxolitinib would be a promising and safe option in newly diagnosed BOS post HSCT.

## Introduction

Characterized by obstructive lung defects and a poor long-term survival rate, bronchiolitis obliterans syndrome (BOS) is a life-threatening pulmonary manifestation of chronic graft-versus-host disease (cGVHD) (Barker et al., 2014). The classic first-line therapy is systemic steroids to prevent progression (Williams et al., 2016). However, during the salvage therapy, when the first-line therapy fails, lots of patients with steroid-refractory BOS did not get a significant improvement in pulmonary function. In addition, long-term systemic steroid application may cause serious complications such as infection and prolonged time of body mass index (BMI) recovery (Hayakawa et al., 2019). We expected to seek for a promising agent, which would be used in combination with systemic steroids, for BOS treatment as first-line therapy at an early stage of pathogenesis, which could be steroid-sparing and save pulmonary function.

Ruxolitinib is a JAK (Janus-activated kinases) 1/2 inhibitor reported to play an important role in the pathogenesis of B cell- and T cell-mediated GVHD, especially to suppress T-cell activation *via* inhibition of cytokine receptor-mediated signaling. (Spoerl et al., 2014) (Zeiser et al., 2015). Moreover, ruxolitinib showed outstanding anti-inflammatory and immunomodulating capacity by affecting dendritic cell (DC) differentiation and function (Heine et al., 2013). It is well-documented that ruxolitinib is an effective treatment option for patients with acute and chronic graft-versus-host disease (aGVHD and cGVHD) in the past decade (Zeiser et al., 2020; Wu et al., 2021). However, there are a few published investigations demonstrating ruxolitinib’s clinical efficacy in treating BOS as a second- or third-line therapy. In this study, we aimed to investigate the effect of ruxolitinib in newly diagnosed BOS post HSCT as a first-line therapy in this study.

## Materials and Methods

All patients who underwent an allogeneic HSCT between January 2019 and December 2021 at the Institute of Hematology and Blood Diseases Hospital, Chinese Academy of Medical Sciences and Peking Union Medical College (CAMS & PUMC) were retrospectively screened. Clinical data were retrieved from the transplant databases and electronic medical records. The inclusion criteria were as referred to the criteria of the 2015 National Institute of Health (NIH) consensus (Jagasia et al., 2015). Measured by pulmonary function tests (PFTs), BOS was defined by having a forced expiratory volume in 1s (FEV1) to a forced vital capacity (FVC) ratio of 0.7 or less, along with FEV1 of <75% predicted with a ≥10% decline over less than 2 years. In addition, expiratory CT or PFTs to support the evidence of air tapping were needed. Infectious diseases should be excluded by clinical and radiological evaluation and routine serological and microbiological studies.

Ruxolitinib therapy began with an initiation dosage of 5 mg twice daily (BID) and then a maintenance dosage of 10 mg. The dose of ruxolitinib could be reduced if severe adverse events occurred, which were graded according to the Common Terminology Criteria for Adverse Events version 4 (CTCAE v4). Steroids and other immune-suppressive agents were added according to the clinical situation. All patients received antifungal prophylaxis and fluticasone, azithromycin, and montelukast (FAM) therapy in addition to ruxolitinib therapy.

Our primary endpoint was the response to treatment, including both symptom response and DR, which were evaluated using PFTs every 3 months. Treatment response included both symptom response (SR) and disease response (DR). Symptom response was defined as relieving respiratory symptoms evaluated using the Lee Chronic Graft-versus-Host Disease Symptom Scale (LSS) (Teh et al., 2020). The assessment was performed 14 days after ruxolitinib administration. Scores <5 were defined as symptom remission, and scores ≥5 were defined as symptoms persisting. We evaluated ruxolitinib DR 3 months after ruxolitinib therapy. The response to ruxolitinib was defined as complete response (CR) when clinical symptoms significantly alleviated and the first–second FEV1 as a percentage of the predicted value (FEV1% predict) increased by more than 75%; partial response (PR) was defined by FEV1% pred levels increased or symptoms improved with stabilization of FEV1% pred. Non-response (NR) was defined by worsened clinical status and PFTs or FEV1% pred decreased down to 5% or less with stable symptoms. Progression disease (PD) was considered when clinical and functional findings (mainly FEV1% pred decreased from baseline by 5% or more) worsened, and stable disease (SD) was considered when FEV1% pred decreased to less than 5% with stable symptoms.

All procedures performed in studies involving human participants were in accordance with the ethical standards of the institutional and national research committee (Ethics committee of Blood Disease Hospital, Chinese Academy of Medical Sciences HG2021042-EC-1). The institutional review board approved all study procedures and forms. Variables of PFTs before and after ruxolitinib were compared between the groups with the chi-square test or Fisher’s exact test. SPSS was used for the statistical analysis (SPSS version 22.0.01; IBM, NY, United States).

## Results

We identified seven patients diagnosed with BOS, and the clinical characteristics are shown in Table 1. Before being diagnosed with BOS, four patients had a history of respiratory infection pneumonia after HSCT. A median time of 272 days (ranging from 57 to 789 days) elapsed between the time of HSCT and diagnosis of BOS. Six patients showed respiratory symptoms such as dyspnea, mild wheezy breathing after activity, or slight cough. The initial symptom of all patients was reduced peripheral oxygen saturation with a median of 93% (range 87%–96%). Once the BOS was diagnosed, ruxolitinib was added at the initiation dosage of 5 mg BID, and three patients used a maintenance dosage of 10 mg BID. Six patients were treated with steroids at the same time. Patient #6 refused to use systemic steroids and took ruxolitinib and FAM only. The initial daily dose of methylprednisolone was 1–2 mg/kg per day. Patient #5 and patient #6 took a calcineurin inhibitor at the same time. The median duration of ruxolitinib application was 121 days (ranging from 90 to 219 days).

**TABLE 1:** Characteristics of seven BO patients.

	Age	Gender	Diagnosis	HSCT Type	Time elapsed between HSCT to BOS (days)	History of pneumonia	SpO2 at diagnosis of BOS (%)	FEV1 at diagnosis (%)	FEV1/FVC at diagnosis (%)	Other organs involved in cGVHD
Patient #1	27	Male	VSAA	HID	103	Yes	87	60.9	60.56	No
Patient #2	19	Male	AML	MSD	124	NO	92	32	60.58	No
Patient #3	11	Female	SAA	HID	489	Yes	96	31	47	Sclerotic-type skin cGVHD
Patient #4	12	Male	SAA	HID	57	No	93	60.2	65	No
Patient #5	49	Female	VSAA	HID	272	Yes	94	44.92	67	No
Patient #6	49	Male	MDS	HID	565	Yes	90	59	61.9	No
Patient #7	23	Male	AML	HID	789	No	90	56.1	53.95	No

	The start of treatment of ruxolitinib after BOS diagnosis (days)	Ruxolitinib maintenance dosage	Ruxolitinib application duration (days)	Methylprednisolone initial dosage	Methylprednisolone application duration (days)	Other second-line drugs	Side effects of ruxolitinib	Symptom response	Disease response
Patient #1	3	10 mg BID	90	2 mg/kg	50	FAM	NA	Symptoms relieved	CR
Patient #2	1	5 mg BID	219	1 mg/kg	58	FAM	Cytopenia	Symptoms relieved	CR
Patient #3	5	10 mg BID	168	1 mg/kg	60	FAM	Pneumonia	Symptoms relieved	PR
Patient #4	1	10 mg BID	146	2 mg/kg	23	FAM	NA	Symptoms relieved	CR
Patient #5	1	5 mg BID	90	2 mg/kg	51	FAM, CSA, and MMF	Cytopenia	Symptoms relieved	CR
Patient #6	1	5 mg BID	121	NA	NA	FAM and FK506	NA	Symptoms relieved	PR

VSAA: very severe aplastic anemia; SAA: severe aplastic anemia; ALL: acute lymphocyte leukemia; AML: acute myelogenous leukemia; HSCT: hematopoietic stem cell transplantation; HID: haplo-identical donor; MSD: matched sibling donor; DR: disease response; CR: complete remission; PR: partial remission; CSA: cyclosporin A; MMF: mycophenolate mofetil; and FK506: tacrolimus.

**FIGURE 1:**
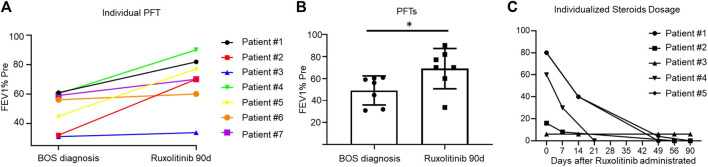
**(A)**: Line graph showing the individualized fluctuation of FEV1% at BOS diagnosis and 3 months after ruxolitinib administration. Figure 1B: FEV1% pred between patients at BOS diagnosis and 3 months after ruxolitinib administration. Figure 1C: A line graph showing the individualized steroids dosing over time during the study period.

Our primary endpoint was the response to treatment, including both symptom response and DR, which were evaluated using PFTs every 3 months. For symptom response assessment, we used the LSS score system. It is inspiring that all patients achieved symptom remission within only 2 weeks after ruxolitinib therapy and were independent of oxygen use (Table 2). For disease response assessment, five patients (71.43%) achieved CR and two (28.57%) achieved PR. The mean FEV1% pred at diagnosis of BOS was 49.16% ± 13.20% (mean ± SD), and it increased to 68.93% ± 18.25% (mean ± SD) 3 months after ruxolitinib therapy (Figures 1A, B).

**TABLE 2:** Evaluation for relieving symptoms 2 weeks after ruxolitinib administration using LSS.

	Patient #1	Patient #2	Patient #3	Patient #4	Patient #5	Patient #6	Patient #7
Frequent cough	0	0	1	0	0	0	0
Colored sputum	0	0	0	0	0	0	0
Shortness of breath at rest	0	1	2	0	2	1	0
Need to use oxygen	0	0	0	0	1	0	0
Fever	0	0	0	0	0	0	0
Total score	0	1	3	0	3	1	0

LSS: Lee Chronic Graft-versus-Host Disease Symptom Scale.

Both patient #3 and patient #6 achieved a PR to the treatment with an increment in FEV1% of 5%. Patient #3 was able to walk continuously for 1 hour independently without wheezing. The FEV1/FVC of patient #6 increased to 59.75% from 53.95%, and he was able to manage his daily life. The therapeutic responses of six patients were continued after a median duration of 7 months, except for patient #5. After 6 months of ruxolitinib treatment, she got a sudden pneumothorax, and thoracic closed drainage was performed. Consequently, she was unable to have any PFTs for response evaluation.

The second endpoint was the therapy course, especially steroid withdrawal. As soon as patients achieved relieving symptoms, we started to perform steroid decrement. Their steroid dose was reduced to 50% of the initial dose after about 2 weeks (range, 7–16 days). Steroid therapy in six patients ended within 2 months (range, 23–58 days) of ruxolitinib treatment (Figure 1C).

In addition, ruxolitinib was safe as a first-line agent (Table 1). Four patients tolerated ruxolitinib without any side effects at the dosage of 5 mg BID. Two patients reduced their ruxolitinib dose from 10 mg to 5 mg due to cytopenia. Patient #3 had a *Streptococcus pneumoniae* infection months after steroid therapy. Overall, no viral and fungal infections were observed in this study.

## Discussion

This retrospective study was targeted to explore the feasibility of ruxolitinib as a first-line therapy in newly diagnosed BOS patients post HSCT. In our study, patients receiving ruxolitinib achieved a remarkable response, including both symptoms relieved and respiratory function improved. Furthermore, the ruxolitinib therapy shortened steroid treatment duration and reduced steroid dosage.

Bronchiolitis obliterans is one of the most common and challenging pulmonary complications post HSCT, with poor long-term prognosis and a 5-year survival rate of only 13%–56% (Grønningsæter et al., 2017). The BO management is generally frustrating in that it leads to airflow obstruction in the majority of patients, and only 8–20% of patients achieve improvement in lung function (Soubani and Uberti, 2007). The pathogenesis of BO was not well-understood. A classic three-phase model has been proposed for the initiation and development of cGVHD, including early inflammation and tissue injury (phase 1), chronic inflammation and dysregulated immunity (phase 2), and aberrant tissue repair often with fibrosis (phase 3) (Cooke et al., 2017). One of the most promising BO pathogenesis mechanisms is alloreactive immune reaction, in which donor T lymphocytes target the bronchioles. In addition, macrophages, B cells, and complicated networks of other cells promoted pulmonary fibrosis (Srinivasan et al., 2012). Systemic corticosteroids and immunosuppressive therapy have been the general management options. In the past decades, several novel options for therapy have emerged, including cyclosporine A, tacrolimus, extracorporeal photopheresis, mycophenolate mofetil, and JAK1/2 and Btk inhibitors.

The efficiency of ruxolitinib in treating cGVHD has been reported. Zeiser et al. (Zeiser et al., 2021) demonstrated that ruxolitinib could lead to a much better overall response in patients with steroid-refractory cGVHD based on their REACH III results. There have been a few studies indicating ruxolitinib’s efficiency in steroid-refractory BOS as a salvage treatment but with controversies. Most investigations approved ruxolitinib’s efficiency in steroid sparing among adult and pediatric SR-BOS patients (Schoettler et al., 2019; Streiler et al., 2020). The underlying mechanism has not yet been well-explicated. One of the promising mechanisms is considered to reduce alloreactive T cell expansion and suppress the activity of macrophages. Moreover, it has been studied that the JAK/signal transducer and activator of the transcription 3 (STAT3) pathway is central to differentiation of B cells into plasma cells and antibody secretion. As an inhibitor of JAK-STAT signaling, ruxolitinib shows efficacy at early stages of inflammation (Schroeder et al., 2018). Otherwise, the effect of ruxolitinib is poor when it comes to the irreversible third phase, which is characterized by fibrosis. This may help explain the results of Bondeelle et al., who claimed ruxolitinib administration did not improve and reverse the course of respiratory function in BOS. (Bondeelle et al., 2020). Zhao et al. (Zhao et al., 2021) analyzed 30 BO patients with ruxolitinib as salvage treatment in their new study, and the best overall response rate reached 66.7%. Their results proposed the administration of ruxolitinib in the early “inflammatory” phase other than the late “fibrotic” phase of BOS pathogenesis. Referring to our study, we found that administration of ruxolitinib in the early stage increased the therapeutic effective rate and prevented BO prognosis significantly, with an ORR of 100% and a CR of 71.43%. We assume that patients could benefit from the application of ruxolitinib as a first-line in newly diagnosed BO other than a salvage treatment. Furthermore, we also suggest intense and frequent routine screening of BO using PFTs and respiratory CT early after HSCT, as early diagnosis and intervention may help BOS outcome improvements.

In our study, there were six patients who also received systemic steroids at an initial dosage of 1–2 mg/kg. It is inspiring that steroid therapy succeeded to end within 2 months. Streiler et al. reported that prednisone dosing decreased by 50% after 3 months of treatment with ruxolitinib as a salvage treatment (Streiler et al., 2020). Compared to the previous study, we found that the combination of ruxolitinib early significantly shortened the steroid usage duration.

In conclusion, introducing ruxolitinib as a first-line treatment in the early pathogenesis of BOS shows the capacity to relieve symptoms rapidly and ameliorate PFTs in our study. It is also noteworthy that we succeeded in shortening the steroid therapy duration to 2 months and reducing the total steroid dosage. In addition, ruxolitinib is well-tolerated, and no severe infection or relapse is reported. Certainly, due to the limited sample size, larger prospective studies are needed to prove that ruxolitinib would be one of the backbones as a first-line treatment in newly diagnosed BOS in the future.

## Data Availability

The raw data supporting the conclusion of this article will be made available by the authors, without undue reservation.
